# Do Integrated Hub Models of Care Improve Mental Health Outcomes for Children Experiencing Adversity? A Systematic Review

**DOI:** 10.5334/ijic.6425

**Published:** 2022-06-17

**Authors:** Suzy Honisett, Hayley Loftus, Teresa Hall, Berhe Sahle, Harriet Hiscock, Sharon Goldfeld

**Affiliations:** 1 Centre for Research Excellence in Childhood Adversity and Mental Health, Centre for Community Child Health, Murdoch Children’s Research Institute, AU; 2 The University of Melbourne, AU; 3 Health Services Research Unit, The Royal Children’s Hospital, AU; 4 Department of Paediatrics, The University of Melbourne, AU; 5 The Centre for Community Child Health, AU; 6 Theme Director Population Health and Co-Group Leader Policy and Equity, Murdoch Children’s Research Institute, AU; 7 Department of Paediatrics, The University of Melbourne, Royal Children’s Hospital, 50 Flemington Rd, Parkville VIC 3052, AU

**Keywords:** integrated care, integrated health service, primary care, child mental health, childhood adversity

## Abstract

This review assesses the effectiveness of integrated primary health and social care hubs on mental health outcomes for children experiencing adversity and describes common integration dimensions of effective hubs.

PubMed, OVID Medline and PyschINFO databases were systematically searched for relevant articles between 2006–2020 that met the inclusion criteria: (i) interventional studies, (ii) an integrated approach to mental health within a primary health care setting, (iii) validated measures of child mental health outcomes, and (iv) in English language.

Of 5961 retrieved references, four studies involving children aged 0–12 years experiencing one or more adversities were included. Most children were male (mean: 60.5%), and Hispanic or African American (82.5%).

Three studies with low-moderate risk of bias reported improvements in mental health outcomes for children experiencing adversity receiving integrated care. The only RCT in this review did not show significant improvements.

The most common dimensions of effective integrated hubs based on the Rainbow Model of Integrated Care were clinical integration (including case management, patient-centred care, patient education, and continuity of care), professional integration, and organisational integration including co-location.

These results suggest hubs incorporating effective integration dimensions could improve mental health outcomes for children experiencing adversity; however, further robust studies are required.

**Registered with Prospero:** CRD42020206015.

## Introduction

Between 10 and 20% of children and adolescents worldwide experience mental health disorders [1,2]. Early intervention is needed to better identify and treat these disorders given that almost half of all lifetime disorders start by 14 years of age and three-quarters by 24 years [3,4]. The distribution of mental disorders is not equal however, with a higher prevalence in children and young people facing adversity. Adversities can include adverse childhood experiences (ACEs) [5,6] i.e., childhood maltreatment (e.g., physical, verbal, or sexual abuse) and household dysfunction (e.g., parental mental illness, family substance abuse) [7], as well as broader social determinants. ACEs intersect with the broader social determinants focusing on where children and families live, work and play, and include community dysfunction (e.g., witnessing physical violence, discrimination) and peer dysfunction (e.g., stealing, bullying) as well as socio-economic deprivation [8]. These adversities can disrupt important mechanisms for development across a child’s life course, including genetic risk, epigenetic modifications, behavioural role modelling, social learning, and relational skills [9]. Adversity has well-established negative impacts on mental health, increasing the risk of anxiety, internalising disorders, depression, and suicidality in childhood and across the life course [10,11,12,13].

Therefore, as a root cause, adversities in childhood are an important target for early intervention given their significant contribution to the disease burden from mental disorders across the lifespan [14]. However, children and families experiencing adversity often do not access evidence-based services in a timely, effective manner [15,16,17]. This is in part due to fragmentation of existing services and a workforce that reports an inadequate understanding of child mental health [18,19]. Integrated health care approaches could effectively address fragmentation, poor access to evidence-based services, and lack of developmentally trained workforce for children and their families [20]. Integrated approaches have been identified by health practitioners as a potential solution to fragmented health care, especially for those most disadvantaged [18].

Primary health care offers an appropriate platform for integrated early intervention. Outlined in the Alma-Ata Declaration [21], primary care provides a strategy of public health derived from a social model of health, making it possible to distribute health services equitably across populations. Primary care also provides first contact and continuous, comprehensive, and coordinated care for families [22,23].

To further add value to the primary health platform, integration is defined as care that: “connect[s] the healthcare system (acute, primary medical and skilled) with other human service systems (e.g., education and vocational and housing services) to improve outcomes (clinical, satisfaction and efficiency)” [24]. The Rainbow Model of Integrated Care (RMIC) provides a useful description of integrated care from the primary care perspective [25]. The RMIC describes integration at the macro- (system integration), meso- (organisational and professional integration) and micro-level (clinical integration). RMIC defines the primary dimensions of integration as clinical integration (i.e., the coordination of person-focused care in a single process across time, place and discipline), professional integration (i.e., inter-professional partnerships based on shared competences, roles, responsibilities and accountability to deliver a comprehensive continuum of care to a defined population), organisational integration (i.e., inter-organisational relationships, including common governance mechanisms, to deliver comprehensive services to a defined population) and system integration (i.e., a horizontal and vertical integrated system, based on a coherent set of (informal and formal) rules and policies between care providers and external stakeholders for the benefit of people and populations). Further, the RMIC identifies supports that enable integration across macro, meso and micro levels, including functional integration (i.e., coordination of financial management, human resources, strategic planning, information management and quality improvement) and normative integration (i.e., mutual shared goals and an integrative culture). Together, these dimensions enable integration between different levels within a primary health care service to ensure the provision of continuous, comprehensive, and coordinated delivery of services to individuals [26].

There has been increased interest in integrated primary health care for early identification and support of child mental health disorders; however, most studies focus on outcomes of access and service quality [27,28]. Few studies have investigated mental health outcomes and even fewer deconstruct the approach of integrated care to understand common elements of care that are most effective [29]. One systematic review [29] in 2020 did focus on child mental health and integrated care but their search was restricted to integrated models of care across health and mental health only, within the United States only, and did not focus specifically on children experiencing adversity. This review seeks to expand upon the Yonek et al. review [29] by focusing on children experiencing adversity and models of care integrating a range of health and social services and evaluated in any country.

### Objectives

Conduct a systematic review of child and family integrated primary care hub models to:

1. Ascertain their effectiveness on child mental health outcomes for children experiencing adversity.
2. Identify integrated primary hub dimensions associated with improvements in child mental health outcomes.

## Methods

This systematic review followed the Preferred Reporting Items for Systematic Reviews and Meta-Analyses (PRISMA) [30] framework. The PICO (population, intervention, control, and outcomes) format [31] was used to develop the research question.

### Literature search

A systematic literature search was undertaken by an experienced research librarian using PubMed, OVID Medline and PsychINFO. The search strategy included a range of MeSH terms and free text to capture studies that focused on integrated mental health care within a primary health care setting for children experiencing adversity from 2006 until 3 August 2020. See Supplement 1 for full details of the search strategy.

Studies were only included if they met each of the following criteria:

1. Included peer-reviewed studies, including randomized controlled trials (RCTs); cluster RCTs; controlled before and after studies where participants are allocated to control and intervention groups using non-randomised methods; interrupted time series studies with before and after measurements; and cost-effectiveness/cost-utility/cost-benefit of integrated primary care hub models.
2. Included children aged 0-12 years experiencing adversity. In the absence of a standardized definition of childhood adversity, we included studies in which children were reported to experience one or more of the following adversities: family violence, parental mental illness, physical, psychological or sexual abuse, child neglect, parental substance misuse, parental incarceration, bullying, harsh parenting, racial minority, indicators of socio-economic disadvantage (e.g., receiving federal government funding for health care such as Medicare/Medicaid (USA only) or welfare payments) [8]. Adversity was considered present if it was explicitly mentioned, regardless of the level of detail reported.
3. Were situated within a primary health care setting. A primary health care setting includes family doctor, general practitioner clinic/s, child health centre, community health service, community mental health service.
4. Included integration within at least two dimensions of the Rainbow Model of Integrated Care (RMIC) [26].
5. Included integrated care that had intersectoral linkages with health and social services. Therefore, we included studies that bridged two or more types of care providers or organisations [32] (GP clinics, allied health teams, maternal and child health, social work, family violence, legal, education, paediatricians, psychologists etc.).
6. Assessed child mental health outcomes (depression, anxiety, externalising or internalising behaviours). We considered validated mental health measures for children above 18 months (e.g., Strength and Difficulties Questionnaire (SDQ) [33], Child Behaviour Checklist [34]). Due to the inconsistency of mental health measures for children under 18 months [35], we considered validated social and emotional development and wellbeing measures (e.g., Ages and Stages Questionnaire – Social and emotional ASQ-SE [36]).
7. Were written in English.

### Study selection

Two authors (SH, HL) screened the same ten percent of titles and abstracts. Discrepancies were identified and resolved, and SH and HL independently screened the remaining 5961 titles and abstracts using Covidence software [37]. Initial screening was followed by an independent review of 126 full-text articles by authors SH, HL and BS. SH reviewed all full-text articles, and discrepancies were resolved between the screening authors.

### Data extraction

One of the authors (SH) independently extracted the following data from the original studies using a standardized data collection form: 1) study characteristics – author, country, year of publication, type of study, participant numbers, control group numbers, study duration; 2) participant characteristics – age, gender, type of childhood adversity experienced; 3) intervention characteristics – setting for the study (e.g., primary care clinic, paediatric medical home), risk of bias; 4) mental health outcomes, and 5) significant results.

One author (SH) coded each study for analysis of integration using the RMIC integration dimensions (i.e., clinical, professional, organisational, system, normative and functional integration) and the related taxonomy of 59 key features for each dimension (e.g., case management, clinical leadership, co-location, good governance etc.) [25]. Integration was classified against relevant dimensions and the key features related to these dimensions [25]. A dimension and key feature of integration were considered present if they were explicitly mentioned, regardless of the level of detail reported.

### Quality assessment

Two authors (SH and TH) independently assessed the methodological quality of the studies. Risk of Bias [38] was used to evaluate the methodological quality of RCTs. The quality of non-randomised studies was evaluated using Risk of Bias In Non-randomised Studies – of Interventions (ROBINS-I) [37]. Discrepancies between reviewers were identified and resolved through discussion.

### Data synthesis

Descriptive statistics were used to summarise the data. Distillation framework, based on previous work by Choprita et al. [39] and Becker et al. [40], was also used to guide data synthesis. Distillation, a qualitative analytical method, allows the synthesis of information from bundled or complex interventions to understand which dimensions are present in an intervention and determine how frequently these occur across studies. The process of distillation has also been used to aggregate which components or combinations of components are associated with study outcomes. In this systematic review, SH employed distillation by classifying each study against the RMIC’s six dimensions and 59 features of integration [25]. These data were then pooled to determine how commonly these dimensions and key features were employed across all studies. Data are presented in Table 2.

SH used the Standard Framework for Levels of Integrated Healthcare [41], developed by Substance Abuse and Mental Health Services Administration and the Health Resources and Services Administration, as a benchmark of the extent of integration. This framework conceptualises integration as a continuum, ranging from separate primary care systems with minimal coordination to integrated systems, and provides a standard classification of integration within a setting. The framework, which is widely used in the United States [39], incorporates common dimensions of integration, as outlined in the RMIC, and categorises these into six levels of integration: Level 1- minimal collaboration; Level 2 – basic collaboration at a distance; Level 3 – Basic collaboration on-site; Level 4 close collaboration on-site with some system integration; Level 5 – Close collaboration approaching an integrated practice; and Level 6 – full collaboration in a transformed/merged integrated practice [40]. Each of these levels includes core descriptions outlining the expected activities and key differentiators across the domains of clinical delivery, patient experience, practice/organisation, and business model. The level of integration for each study was rated against these core descriptions and differentiators, presented in Table 2.

## Results

### Participant and study characteristics

**Figure 1:**
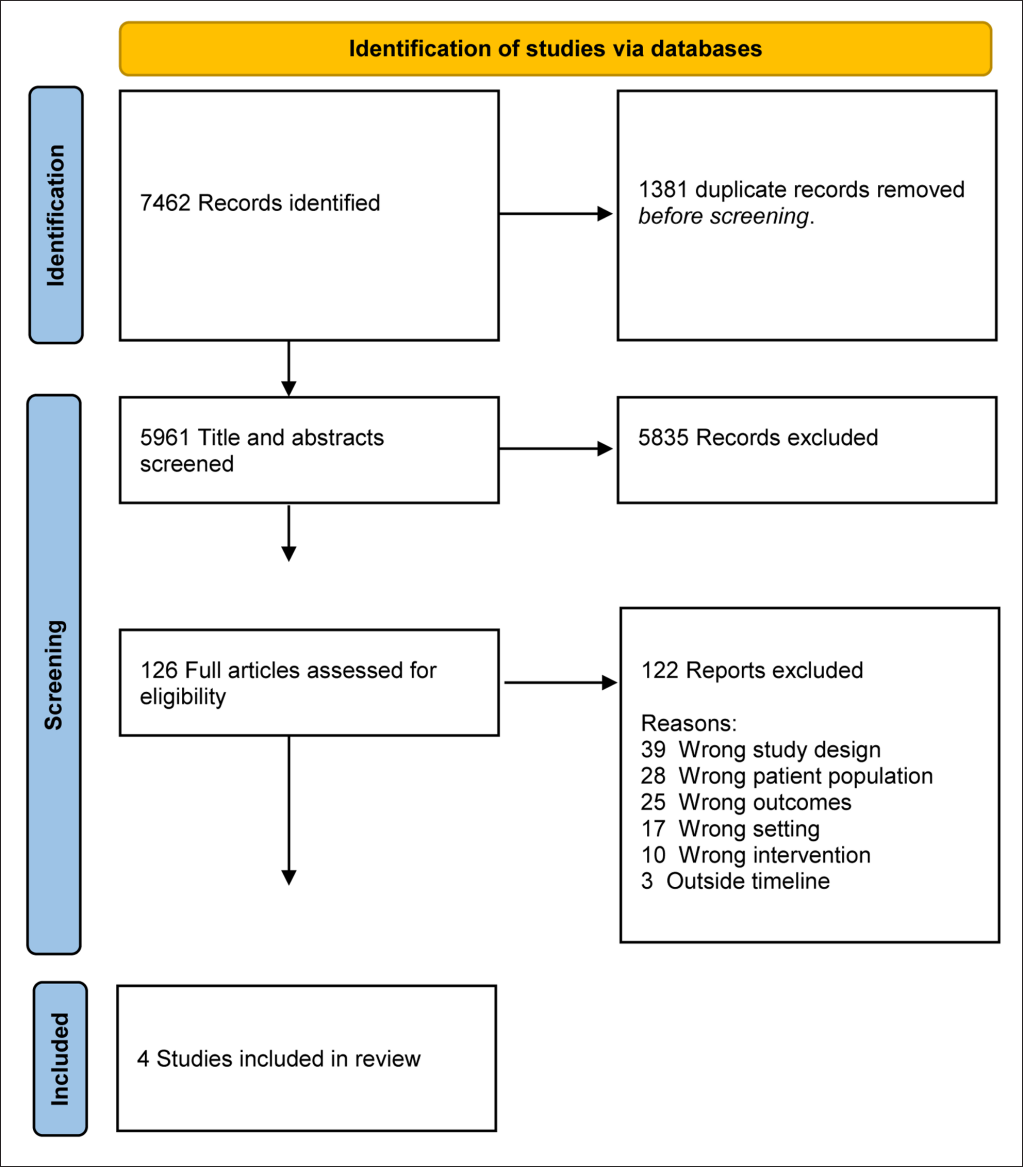
PRISMA Flow diagram of screening results.

The search retrieved 7,462 articles. After excluding duplicates, 5961 records were screened for eligibility. Four studies met all inclusion and exclusion criteria and were included in this systematic review. See Figure 1 for PRISMA flow diagram [30] of the results of the screening process.

The included studies were methodologically heterogenous. One study was a RCT including 99 children [42], one a pre-post study of 116 children with attention deficit hyperactivity disorder (ADHD) [43], and two studies were prospective cohorts – one involving 118 children assessed at baseline, 6- and 12-months [44] and the other involving 79 children with elevated ASQ-SE measures [45]. The length of most studies ranged from 12 to 18 months. The Briggs et al. [45] cohort study was undertaken for 5 years. See Table 1 for study characteristics.

**Table 1:** Characteristics of the four included studies.


AUTHOR/YEAR/LOCATION	*STUDY CHARACTERISTICS*						*PARTICIPANT CHARACTERISTICS*			*OUTCOME CHARACTERISTICS*		*RISK OF BIAS (MEASURE USED)*
	
	*TOTAL SAMPLE*	*INTERVENTION GROUP*	*CONTROL GROUP*	*STUDY TYPE/DURATION*	*SETTING*	*INTERVENTION*	*AGE MEAN (YEARS)*	*SEX (%)*	*ADVERSITY*	*OUTCOME MEASURES*	*SIGNIFICANT OUTCOMES*	

Briggs et al. 2011 (United States)	79 with elevated ASQ:SE scores	41	38	Prospective cohort 5-year duration	Primary care paediatric practice	Children identified with elevated ASQ:SE scores through universal screening were offered evaluation by Infant Toddler Specialists and appropriate treatment or referral in consultation with paediatric provider. Treatment included education and family support.	47.4% 0-12 months Mean and SD not available (range 6 months -3 years)	52% Male	Racial minority – Hispanic or African American – 82.1% Receiving Medicaid or other state-sponsored insurance programs – 68.9%	ASQ-SE	**ASQ-SE** Significant improvement on ASQ:SE scores in intervention children compared with those who declined intervention (p = .01)	Low (ROBINS-I)

Molnar et al. 2018 (United States)	225	225	–	Prospective cohort 12 months	Paediatric medical homes at 3 sites servicing primarily low-income residents	Early Childhood Mental Health clinician and family partner (case manager) provided: case coordination with paediatric medical homes, care planning, referrals as needed and child mental health and or parenting interventions.	3.26 years (SD 2.01, range 0-8 years)	62% Male	Racial minority African American – 34% Hispanic – 53%	ASQ-SE for children aged 5 years and younger CBCL for children aged 6-8 years	**ASQ – SE** Significant declines in social, emotional and behavioural problems for children under 5 years Children who started above the clinical cut-off score on average scored below (in the healthy range) by timepoint 3 (p < 0.0001) **CBCL** Those who scored above clinical cut-off scores at baseline had a 37% decline at time 3 (p < 0.001).	Low (ROBINS-I)

Myers et al. 2010 (United States)	116 diagnosed with ADHD	116	–	Pre/post-intervention Monthly reviews until 14 months or until stable	2 paediatric clinics (1 rural, 1 urban)	A Care Manager liaised between treating physicians and consulting psychiatrists to develop and implement care plan related to treatment and basic parent education.	8.84 years (SD 1.99, range 6-12 years)	73% Male	Racial minority – Hispanic – 95%	VADPRS and VADTRS	**VADPRS – ADHD**, oppositional defiant disorder, and conduct disorder symptom and performance subscales improved significantly (P < .05). **VADTRS** – symptom subscales improved (p < 0.001) but performance subscales did not.	Moderate (ROBINS-I)

Wansinket al. 2015 (Netherlands)	99	49	50	RCT 18 months	Community mental health centre	The Preventative Basic Care Management (PBCM) program provided parents case management to design a tailored plan for the family, link families to evidence-based parenting strategies, home-based family support, psychoeducation, community health services, social services, services for debt restructuring and financial resources. Also provide care coordination. Control: Parents received a brochure about the impact of parenting problems on children and information about available services.	6.08 years (SD 2.02, range 2.3–10.7 years)	56% Male	Parent with a mental illness – (depression 39%, PTSD 15%, anxiety disorder 13%). Single parent – 46% Racial minority – 67% Receiving Medicaid or other state-sponsored insurance programs – 38%	SDQ	**SDQ** No significant effect on SDQ	Low- Moderate (Risk of Bias)


*ASQ-SE* – Ages and Stages Questionnaire – Social and Emotional. *CBCL* – Child Behaviour Check List. *VADPRS* – Vanderbilt ADHD Parent Rating Scales. *VADTRS* – Vanderbilt ADHD Teacher Rating Scales. *SDQ* – Strengths and Difficulties Questionnaire.

A total of 519 children participated in the included studies. All studies included children aged 0-12 years; however, the prospective cohort [44] included younger children aged 0-3 years. Most child participants were male (mean [SD], 60.5% [8%]). For three studies that were based in the United States, most participants were either Hispanic or African American (mean [SD], 82.5% [10%]). Childhood adversity differed across all studies (see Table 1).

Studies took place in a primary care paediatric practice [45], paediatric medical homes [43,44], or a community mental health centre [42].

Two studies were rated to have a low risk of bias [44,45] and two studies were rated to have a low-moderate risk of bias [42,43]. Inability to blind participants and health service providers to the intervention provided and use of parent reported outcome measures were the most common potential sources of bias.

The studies included in this review were heterogenous in terms of methodology, interventions, and outcomes; therefore, a meta-analysis was not considered appropriate.

### Child mental health outcomes

Three non RCT studies demonstrated improved mental health outcomes of children experiencing adversities [43,44,45]. Two studies used ASQ-SE and observed statistically significant improvements in scores (p < 0.0001 – 0.01) [44,45]. The study including children with ADHD saw significant improvements in oppositional defiant disorder and conduct disorder symptoms and performance subscales (p < 0.05) [43]. The RCT, which included children experiencing the highest number of adversities (parents with a mental illness, single parent families, families belonging to ethnic minorities) and used intention to treat analysis, found no significant improvement in child mental health outcomes measured by the SDQ [42].

### Integration characteristics

All studies included at least two dimensions of integration and included care across two or more types of care providers or organisations (see Table 2).

**Table 2:** Dimensions of integration incorporated within the four included studies.


AUTHOR	LEVEL OF INTEGRATION*	DIMENSIONS OF INTEGRATION BASED ON RMIC AND KEY COMPONENTS	EXAMPLES OF INTEGRATION ACROSS THE RMIC WITHIN THE STUDY

Briggs et al. 2011	4	Clinical integration *Centrality of client needs* *Case management* *Continuity* *Information provision to clients*	The intervention coordinated high-quality social and emotional screening, complete with follow-up assessment and intervention referral or support Children who screened above the ASQ:SE risk cut-off thresholds were referred for assessment/intervention to the case manager – Infant Toddler Specialist (ITS), which enabled monitoring, on-site intervention, or referral depending on clinical evaluation An information letter (Spanish and English) was provided to families about the purpose of screening

		Professional integration *Agreement on interdisciplinary collaboration*	The Infant and Toddler Specialist (ITS) made treatment and referral decisions in consultation with paediatric provider

		Organisational integration *Location policy*	Co-location of bilingual early childhood mental health professionals directly in the paediatric primary care medical home

Molnar et al. 2018	4	Clinical integration *Centrality of client needs* *Case management* *Patient education* *Continuity* *Interaction between professionals and client*	Case manager was a ‘family partner’ with lived experience raising a child with a history of social, emotional or behavioural difficulties to work collaboratively with families drawing on shared experiences and role modelling effective strategies ‘Family partners’ worked collaboratively with clinicians who had masters-level training in mental health care for very young children Initiation of case management and related referrals; and, as needed, child mental health and/or parenting interventions

		Professional integration *Shared vision between professionals* *Inter-professional education*	Collaborative development of a care plan based on child needs and family priorities Teams benefitted from cross-site/cross-project learning collaboratives and monthly meetings with medical and behavioural staff from each site

		Organisational integration *Learning organisations* *Location policy*	Team members participated in on-going training run jointly by local and state health departments on evidence-based early childhood development, mental health and parenting interventions

		Functional integration *Human resources management*	‘Family partners’ were employed by the health care sites Clinical consultation, technical assistance and administrative supervision was provided by the local public health team throughout to assist in integration of intervention services into each centre and in keeping fidelity to the model

Myers et al. 2010	2–3	Clinical integration *Case management* *Client satisfaction* *Patient education*	Case manager liaises between treating physician and psychiatrist Families were interviewed on their experience and improvement recommendations Patients were educated about the aetiology and management of ADHD

		Organisational integration *Location policy*	Case manager was co-located with paeditricians at one site

Wansink et al. 2015	2	Clinical integration *Case management* *Continuity*	Broker model of case management used Organisation of care aimed to provide fluid care delivery by linking with psychiatric and preventive services

		Professional integration *Shared vision between professionals*	Case manager contacts the family and services to evaluate goals and arrangements


* Level 1– minimal collaboration; Level 2 – basic collaboration at a distance; Level 3 – Basic collaboration on-site; Level 4 close collaboration on-site with some system integration; Level 5 – Close collaboration approaching an integrated practice; and Level 6 – full collaboration in a transformed/merged integrated practice [39].

Although studies were heterogeneous in design and delivery, clinical integration was present in all studies and professional and organisational integration dimensions were present in 75% of studies.

#### Clinical integration – the extent to which person-focused care is coordinated

##### Case management

Case management aims to coordinate care by bringing together professionals from different sectors and families to identify risks, establish care plans and monitor child and family progress [25]. Although case management was identified in all four studies, its application varied across studies. Molnar et al. [44] employed a ‘family partner’ with lived experience raising a child with a history of social, emotional, or behavioural difficulties as a case manager. The ‘family partner’ engaged families by drawing on shared experiences, modelled effective strategies for parenting and worked collaboratively with clinicians. Briggs et al. [45] employed an Infant Toddler Specialist case manager who was a licensed bilingual early childhood psychiatrist. Wasnick et al. [42] employed a broker model of case management, whereby the broker supported the family to identify their needs and brokered supportive services in one or two contacts [46]. This model assumed that a caregiver knowledge of service options and access pathways would increase service use.

##### Continuity

Continuity – the organisation of care to provide ongoing and appropriate care delivery for children and families [25], was present in three studies [42,44,45]. These three studies consistently implemented the following continuity processes: child assessment, identification of risk, intervention, or referral, and follow up to provide a continuous flow of care. However, the quality of application or implementation was not measured and may vary between studies.

##### Patient education

Parent education was included in two studies [43,44] to varying levels. Molnar et al. [44] used role modelling of effective parenting strategies to educate parents, while Myers et al. [43] provided parent education about the aetiology and management of ADHD.

#### Professional integration – the sharing of roles, competencies and responsibilities

Professional integration was identified in three studies, although the number of ways this dimension was implemented was lower than that for clinical integration (3 vs. 7, respectively, see Table 2). The key features of this dimension were spread across three areas -inter-professional education, shared vision between professionals, and agreement on interdisciplinary collaboration. Having a shared vision between professionals, whereby the shared vision focused on the patient care provided, was consistent for two studies [42,43].

#### Organisational integration – collaboration through contracting and alliance

##### Co-location

Organisational integration was included in three studies [43,44,45] with co-location of health, mental health and social care professionals at study sites. Briggs et al. [45] co-located a child psychologist to operate as a case worker with developmental and behavioural paediatricians, physicians, social workers, nurses, and a nutritionist. Myers et al. [43] co-located social work trained case managers with paediatricians in one of its two sites. Molnar et al. [44] co-located ‘family partner’ case managers with masters-level training in mental health care for very young children, with primary care physicians, nurses, and social workers.

### Components of integration related to improved child mental health outcomes: distillation analysis findings

Three studies [43,44,45] reported a positive association between an integrated model of care and significant improvements in clinical outcomes (symptom severity). All three studies with significant improvements in mental health outcomes included the dimension of clinical integration. Within this dimension, the key integration features that were frequently identified were case management [43,44,45], care focusing on client medical, psychological, and social needs [45], patient education [44,45] and continuity [44,45].

Two studies with significant improvement in mental health outcomes also included professional integration [44,45]. Studies employed interprofessional education [44], or shared vision between professionals [44] or agreement on interdisciplinary collaboration (n = 1) [45].

Organisational integration was included in all studies that reported significant mental health outcomes. Within this dimension of integration, co-location was the key feature.

#### Extent of integration

Using the Standard Framework for Levels of Integrated Healthcare [40], studies were benchmarked against criteria for each level of integration within the framework. Two studies were rated low-to-moderate levels of integration – Myers et al. [43] rated Level 2–3, indicating basic collaboration, and Wasnick et al. [42] rated Level 2 – basic collaboration at a distance (no co-location). Briggs et al. [45] and Molnar et al. [44] were rated as achieving a high level of integration – Level 4, which represented close collaboration onsite with some system integration.

## Discussion

This review suggests that integrated Hubs, based in primary care settings, may be an appropriate model of care to improve child mental health outcomes in children experiencing adversity. Effective components of integrated Hubs likely include clinical integration (including case management, patient-centred care, patient education, and continuity of care), professional integration, and organisational integration including co-location.

Despite heterogeneity in study methodology, level of integration and mental health outcome measures, the review identified three studies of integrated health and social care Hubs that were associated with improved mental health outcomes. The most rigorously designed study – an RCT, did not show a significant effect of integrated care on child mental health outcomes. However, there are three important points to consider in relation to the RCT. Firstly, a sample size calculation was not published, and this trial was potentially underpowered. Secondly, the RCT did find a significant interaction effect of time and intervention on parenting skills and a trend toward improvements in parenting quality. This is an important proximal outcome as child mental health is influenced by parenting quality and skill [47]. Thirdly, this study included children with at least three adversities, including parents treated for a psychiatric illness, compared to other studies in this review where families were experiencing one to two adversities. This suggests that families experiencing multiple adversities may require further intensive support over a longer period to improve child mental health outcomes and aligns with a wealth of literature showing the cumulative negative impacts of adversity [48]. These three points should be considered when assessing the overall effectiveness of studies within this review.

Although integrated care within these studies included a complex bundling of interventions, a key strength of this review was our use of distillation methods to identify the integration dimensions and key features associated with improved mental health outcomes for children. Dimensions of clinical, professional, and organisational integration were common across all studies. Models of care targeted at children experiencing adversity may consider incorporating these micro and meso levels of integration to achieve better child mental health outcomes, recognising that most health and social problems are interrelated [26]. Functional integration, which supports clinical, professional, organisational and system integration, was only identified in one study, while no studies included system integration. The lack of functional and system integration within the reviewed studies may indicate that clinical, professional, and organisational integration may be easier to achieve, serving as a starting point to establish an enabling environment before greater levels of integration can be achieved.

Common features of integration identified in studies associated with improved outcomes were case management and co-location and, to a lesser degree patient education, continuity of care, and patient-centred care. These may provide a foundation for integration on which primary care settings can build further effective practice. This is consistent with a review by Yonek et al. [29] that showed case management was present in all studies that reported significant improvement in patient satisfaction, health-related quality of life, and care quality. Several practices may be central to good case management, including assessment, coordination of and referral and linkage to services, case monitoring and planning, development of individualised plans, and provision of information, education support and direct services [49]. Co-location, has also been shown in adult studies to contribute to greater patient access, cooperation, and collaboration between services [50,51]. Co-location provides opportunities for collaboration; however, it does not necessarily guarantee collaboration [50].

A key strength of this review includes the use of an existing and well-established framework for integration, the RMIC, to evaluate the dimensions of integration included in these complex and bundled interventions. This adds to our knowledge of what to integrate and how, to achieve improved mental health outcomes for children. Other strengths of this review include a comprehensive search of the literature, utilising two independent reviewers to assess studies for inclusion, and undertaking a robust assessment of bias.

This review had several limitations. Firstly, we were unable to conduct a meta-analysis due to the heterogeneity between studies. Secondly, our review may have been impacted by a lack of common and consistent terminology for integrated primary care, potentially limiting the number of studies identified in the initial search. However, we identified over 7000 papers, suggesting that our search terms covered a large number of studies. There was often little detail within studies about integration components and how these were implemented. This may have reduced the range of integration strategies and activities we could identify within the included studies, making it challenging to link mental health outcomes to a full suite of integration features. A third limitation to this review was the focus on integrated health and social care models within primary care settings. There is a growing body of evidence on the effect of integrated care on child mental health in other settings, including but not limited to early childhood services [52,53] and primary schools [54,55]. These settings each provide an ideal platform to engage a wide population of children and their families to identify and respond to emerging mental health issues early.

The results of this review suggest that integrated care Hubs within primary care may be a promising approach to improve child mental health outcomes. The seemingly more effective integration dimensions identified in this review may prove useful starting points for implementation.

The dearth of robust study designs evaluating the outcomes of integrated Hubs requires further research to ensure optimal investment of the public dollar. This includes more rigorous evaluation to establish if integrated Hubs are truly effective (in terms of improved outcomes), lead to more equitable access, and are cost-effective and sustainable from healthcare and societal perspectives. Future research is also required to establish which components of integrated care are associated with improved child mental health, and for which populations. However, designing and conducting research to tease out which components of integrated care are effective and for which populations will be challenging. More traditional RCT designs (including sequential, multiple assignment, randomized trials (SMARTs)) [56] may not be implementable in these populations. Instead, evaluations that acknowledge complexity of interventions and instead seek to understand what works, for whom, under what circumstances and how – as per realist evaluation [57], might be more suitable.

## Conclusion

This review offers promising evidence for the concept of integrated primary health and social care hubs for children and families as an early intervention approach to improve child mental health. Embedding an integrated approach within primary care settings may provide an equitable service delivery platform for early intervention. Key components of effective integrated care models are likely to include clinical integration as a starting point to integration, particularly case management, patient-centred care and patient education, and ideally co-location where possible. Other dimensions of integrated care may further strengthen this approach but require further studies.

## Additional File

The additional file for this article can be found as follows:

10.5334/ijic.6425.s1 Supplement 1. Search strategy.
