# Supplementary material for: Do Integrated Hub Models of Care Improve Mental Health Outcomes for Children Experiencing Adversity? A Systematic Review
Source: Int J Integr Care. 2022 Jun 17;22(2):24. doi: 10.5334/ijic.6425 (PMC9205372; doi:10.5334/ijic.6425)
Supplement: Supplement 1. — Search strategy. [file ijic-22-2-6425-s1.pdf]

Supplement 1.

Search strategy

#1 Title/Abstract
((“Behavior*” OR “behaviour*” OR “hyperactivity” OR “hyper-activity” OR “inattention” OR “impulsive*” OR “oppositional*” OR “social-skills” OR “anti-social” OR “antisocial” OR “conduct” OR “development*” OR “neurodevelopment*” OR “mood”) AND (“disorder*” OR “problem*”)) OR “adhd” OR “depress*” OR “anxiety” OR “attention-deficit”
#2 Title/Abstract
(“internalising” OR “internalizing” OR “externalising” OR “externalizing”) AND (“behavior*” OR “behaviour*”)
#3 Title/Abstract
“treat*” OR “therap*” OR “diagnos*” OR “prevent*” OR “train*” OR “teach*” OR “program*” OR “interven*” OR “educat*”
#4 (#1 OR #2) AND #3
#5 Title/Abstract
“Interprofessional-relation*” OR “*interdisciplinary-communication” OR ((“integrat*” OR “enhanced” OR “coordinat*” OR “co-ordinat*” OR “collaborat*” OR “individual*” OR “interdisciplin*” OR “inter-disciplin*” OR “multidisciplin*” OR “multi-disciplin*” OR “multiprofessional*” OR “multi-professional*” OR “multiagenc*” OR “multi-agenc*” OR “multi-sector*” OR “cooperative” OR “co-operative” OR “inter-professional*” OR “interprofessional*” OR “intersectoral” OR “inter-sectoral” OR “intrasectoral” OR “intra-sectoral” OR “linked” OR “shared” OR “transition*” OR “continuity” OR “continuum” OR “managed” OR “comanage” OR “co-manage” OR “colocate” OR “co-locate”) AND (“care” OR “team*” OR “system*” OR “service*”)) OR “patient-care-planning” OR “patient-care-team*” OR “patient-care-management” OR “treatment-planning”
#6 Title/Abstract
“Linkage*” OR “care-pathway*” OR “horizontal-integration” OR “vertical-integration” OR “longitudinal-integration” OR “virtual-integration” OR “service-network” OR “whole-system-thinking” OR “disease-manage*” OR “case-manage*” OR “care-manage*” OR “care-plan*” OR “self-care” OR “hub” OR “hubs” OR “refer*” OR “consult*” OR “telemedicine” OR “tele-medicine” OR “telehealth” OR “tele-health”
#7 Title/Abstract
“primary-health*” OR “primary-care” OR “general-practice” OR “general-practitioner*” OR “family-practice” OR “family-practitioner*” OR “family-physician*” OR “family-medicine”
#8 Title/Abstract
(“patient” OR “client” OR “family” OR “person” OR “people”) AND (“centred” OR “centered” OR “focused”)
#9 #5 OR #6 OR #7 OR #8
#10 Title/Abstract
“effect*” OR “Outcome*” OR “Access*” OR “Improve*” OR “quality-of-care”
#11 Title/Abstract
“Domestic-violence” OR “abuse*” OR “rape*” OR “adverse-experience*” OR “traumatic-experience*” OR “battered” OR “neglect*” OR “maltreat*” OR “mistreat*” OR “molest*” OR “physical-punish*” OR “physically-punish*” OR “violence” OR “assault” OR “torture*” OR “adverse-trauma*” OR “early-life-trauma” OR (“parent*” OR “parental” OR “father*” OR “paternal” OR “mother*” OR “maternal” OR “care-giver*” OR “caregiver*” OR “guardian*”) AND (“absence” OR “loss” OR “death*” OR “dying” OR “incarcerat*” OR “prison*” OR “jail*” OR “divorce*” OR “separation” OR “mentally-ill” OR “mental-ill*” OR “depression” OR “depressive-disorder*” OR “drug-use” OR “drug-abuse” OR “addict*” OR “mood-disorder*” OR “bipolar-disorder*” OR “dysthymic-disorder*”)

#12 Title/Abstract
“newborn*” OR “new-born*” OR “baby” OR “babies” OR “neonat*” OR “neo-nat*” OR “infan*” OR “toddler*” OR “pre-schooler*” OR “preschooler*” OR “kinder” OR “kinders” OR “kindergarten*” OR “kinder-aged” OR “boy” OR “boys” OR “girl” OR “girls” OR “child” OR “children” OR “childhood” OR “pediatric*” OR “paediatric*” OR “school-age*” OR “schoolage*” OR “school-child*” OR “schoolchild*” OR “school-girl*” OR “schoolgirl*” OR “school-boy*” OR “schoolboy*”

#13 **All fields**
NOTNLM OR publisher[sb] OR inprocess[sb] OR pubmednotmedline[sb] OR indatareview[sb] OR pubstatusaheadofprint

#14 #4 AND #9 AND #10 AND #11 AND #12 AND #13
